# Convolutional neural networks for medical imaging in resource-constrained settings: a scoping review of architectures, performance, and deployment challenges

**DOI:** 10.3389/frai.2026.1894126

**Published:** 2026-09-09

**Authors:** David Chinaecherem Innocent, Rejoicing Chijindum Innocent, Increase Praise Innocent

**Affiliations:** 1 Machine Learning and Computational Research Cluster, Centicini Research Lab, Centicini, Abuja, Nigeria; 2 Department of Epidemiology, School of Public Health, University of Port Harcourt, Port Harcourt, Rivers, Nigeria

**Keywords:** artificial intelligence, convolutional neural networks, deep learning, diagnostic performance, medical imaging, resource-constrained settings

## Abstract

**Background:**

Convolutional neural networks (CNNs) have emerged as powerful artificial intelligence tools for medical image analysis, demonstrating substantial improvements in disease detection, classification, and diagnostic support. Despite increasing evidence regarding their clinical performance, implementation within resource-constrained healthcare environments remains challenging due to limitations in infrastructure, computational resources, dataset availability, and healthcare workforce capacity. Understanding the current evidence relating to CNN architectures, performance characteristics, and deployment barriers is necessary to support sustainable implementation in underserved healthcare settings.

**Aim:**

This review aimed to critically map the available evidence regarding convolutional neural networks for medical imaging in resource-constrained settings, with emphasis on CNN architectures, diagnostic performance, and deployment challenges.

**Methods:**

This scoping review followed the Preferred Reporting Items for Systematic Reviews and Meta-Analyses Extension for Scoping Reviews (PRISMA-ScR) guideline. Literature searches were conducted across PubMed, Scopus, Web of Science, IEEE Xplore, and ScienceDirect from database inception to April 2026. Search terms combined Medical Subject Headings (MeSH) and free-text keywords using Boolean operators. Eligibility criteria were developed using the population–concept–context (PCC) framework. Peer-reviewed English-language studies investigating CNN applications in medical imaging relevant to resource-constrained healthcare settings were included. Data extraction and synthesis were performed thematically.

**Results:**

A total of 18 studies met the inclusion criteria. Five major themes were identified: diagnostic accuracy and clinical performance of CNN models; lightweight and resource-efficient CNN architectures for low-resource deployment; explainability and interpretability of CNN systems; transfer learning and optimization for limited datasets; and implementation barriers affecting deployment in resource-constrained settings. CNN systems demonstrated strong diagnostic performance across tuberculosis, pneumonia, malaria, skin cancer, and other imaging applications, with several studies reporting near expert-level performance. Lightweight architectures and transfer learning approaches improved computational feasibility, while explainability methods enhanced transparency and clinician confidence. However, implementation challenges including computational limitations, poor connectivity, dataset scarcity, and algorithmic bias persisted across studies.

**Conclusion:**

CNN-based medical imaging systems demonstrate considerable potential for strengthening diagnostic capacity within underserved healthcare environments. Nevertheless, sustainable implementation requires greater emphasis on context-specific model development, explainability, locally representative datasets, and real-world deployment studies to ensure equitable and effective adoption in resource-constrained healthcare systems.

## Introduction

Convolutional neural networks (CNNs) are a class of deep learning algorithms specifically designed to process image data through hierarchical feature extraction, enabling automated recognition of complex visual patterns that are often imperceptible to the human eye (LeCun et al., 2015). Within medical imaging, CNNs have transformed diagnostic workflows by improving image classification, segmentation, lesion detection, and disease prediction across modalities such as computed tomography (CT), magnetic resonance imaging (MRI), ultrasound, chest radiography, dermoscopy, and histopathology (Zhou et al., 2021; Wang S. et al., 2021). The rapid integration of CNNs into healthcare has been driven by the increasing global burden of diseases requiring imaging-supported diagnosis. The World Health Organization estimated that nearly two-thirds of the global population lacks access to basic diagnostic imaging services, with shortages disproportionately concentrated in low- and middle-income countries (LMICs) where radiologist density may fall below one specialist per million population (World Health Organization, 2023). Sub-Saharan Africa alone carries approximately 24% of the global disease burden but possesses less than 3% of the global healthcare workforce, creating significant diagnostic delays and preventable mortality (The Lancet Global Health, 2021). Several studies have demonstrated that CNN-based systems can achieve diagnostic performances comparable to expert clinicians in selected tasks. For example, Esteva et al. (2017) reported dermatologist-level classification of skin cancer using deep CNNs, while Rajpurkar et al. (2017) achieved high diagnostic accuracy for pneumonia detection from chest X-rays. More recent evidence indicates that CNN models for lung nodule classification have achieved accuracies exceeding 94%, sensitivities above 92%, and specificities approaching 97%, highlighting their expanding clinical reliability (Desai et al., 2024). Nevertheless, despite these advances, the translation of CNNs from experimental settings into resource-constrained healthcare environments remains inconsistent and poorly synthesized within current literature.

The growing interest in artificial intelligence (AI)-assisted diagnostics in LMICs reflects the urgent need to compensate for limited imaging infrastructure, workforce shortages, delayed laboratory confirmation, and restricted specialist access (Mienye et al., 2025). Resource-constrained settings are generally characterized by inadequate computational infrastructure, unstable electricity supply, low internet penetration, poor interoperability systems, and constrained healthcare financing, all of which directly affect the deployment feasibility of deep learning systems (Kornaros, 2022). Although sophisticated CNN architectures such as ResNet, DenseNet, EfficientNet, U-Net, and MobileNet have shown impressive performance in high-resource clinical environments, many require extensive computational power, large annotated datasets, and cloud-based inference systems that are often impractical in rural or underfunded healthcare systems (Zhou et al., 2021). Critical concerns also persist regarding algorithmic bias, particularly because many CNN models are trained predominantly on datasets derived from populations in North America, Europe, and China, limiting transferability to African, South Asian, and other underrepresented populations (Seyyed-Kalantari et al., 2021). Studies have further argued that the overemphasis on accuracy metrics without sufficient attention to deployment realities creates a misleading perception of readiness for clinical adoption in LMICs (Wang S. et al., 2021). For instance, models reporting exceptional diagnostic accuracy may still fail in real-world settings due to poor-quality images, inconsistent lighting, low-resolution devices, and absence of internet-enabled computational support. Furthermore, explainability remains a persistent challenge, as clinicians frequently express reluctance to trust “black-box” AI systems lacking transparent reasoning mechanisms (Tjoa and Guan, 2020). This limitation becomes even more significant in fragile health systems where diagnostic accountability and medico-legal clarity are essential. Consequently, the literature increasingly suggests that lightweight and explainable CNN architectures capable of offline deployment may represent more practical solutions for underserved settings than computationally intensive state-of-the-art models (Nguyen and Hy, 2026).

Despite the rapid expansion of CNN-related medical imaging research, substantial fragmentation exists regarding how architectures, diagnostic performances, and deployment barriers are collectively analyzed within resource-constrained environments. Existing reviews have predominantly focused on technical performance comparisons across imaging modalities or disease groups while paying limited attention to contextual implementation realities in LMICs (Wang S. et al., 2021; Esteva et al., 2017). Several reviews discuss CNN evolution broadly but fail to critically evaluate infrastructure dependence, energy efficiency, hardware optimization, fairness across skin tones or populations, explainability mechanisms, and offline usability, all of which are central determinants of sustainable deployment in low-resource healthcare systems. Moreover, there remains limited synthesis regarding whether lightweight architectures such as MobileNet or EfficientNet genuinely provide clinically acceptable trade-offs between computational efficiency and diagnostic performance in real-world frontline healthcare contexts. Current evidence also demonstrates inconsistency in evaluation metrics, dataset representativeness, and external validation practices, raising concerns regarding generalizability and authenticity of reported findings across diverse populations. Importantly, no recent scoping review has comprehensively mapped CNN architectures alongside deployment feasibility, operational constraints, and implementation challenges specifically within resource-constrained healthcare settings. Addressing this gap is increasingly important as governments, global health agencies, and researchers continue to advocate AI-enabled diagnostics as potential solutions for strengthening healthcare access and disease surveillance in underserved regions. Unlike previous reviews that primarily summarize CNN performance across disease categories or imaging modalities, this review specifically maps the interaction between CNN architectures, deployment feasibility, explainability, transfer learning, and implementation barriers within resource-constrained healthcare systems. To our knowledge, this is the first scoping review to comprehensively integrate CNN architecture selection, deployment feasibility, explainability, transfer learning strategies, and implementation barriers specifically for medical imaging in resource-constrained healthcare settings. Therefore, this scoping review aims to critically map the existing evidence on convolutional neural networks for medical imaging in resource-constrained settings, with emphasis on architectures, diagnostic performance, and deployment challenges.

## Methods

This scoping review followed the Preferred Reporting Items for Systematic Reviews and Meta-Analyses Extension for Scoping Reviews (PRISMA-ScR) guideline to ensure methodological transparency, reproducibility, and comprehensive reporting of the review process (Tricco et al., 2018). The review methodology was designed to systematically map the breadth of evidence relating to CNNs for medical imaging in resource-constrained settings, with particular emphasis on model architectures, diagnostic performance, implementation feasibility, and deployment challenges. A scoping review approach was considered most appropriate because the field of artificial intelligence-assisted medical imaging remains rapidly evolving, multidisciplinary, and methodologically heterogeneous, thereby requiring broad evidence mapping rather than narrow effectiveness synthesis (Arksey, 2005). The methodological process was further informed by recommendations from Levac et al. (2010), particularly regarding iterative searching, study selection refinement, and thematic synthesis of emerging evidence.

### Search strategy

A comprehensive and structured literature search was conducted across five major electronic databases, including PubMed, Scopus, Web of Science, IEEE Xplore, and ScienceDirect, to identify peer-reviewed studies relevant to CNN-based medical imaging in low-resource and underserved healthcare environments. The databases were searched from inception until 30 April 2026 to maximize retrieval of both foundational and contemporary studies within the field. The search strategy combined Medical Subject Headings (MeSH) terms with free-text keywords and synonymous terminologies relevant to convolutional neural networks, deep learning, medical imaging, artificial intelligence, diagnostic imaging, resource-constrained settings, low- and middle-income countries, and deployment challenges. Boolean operators including “AND,” “OR,” and “NOT” were systematically employed to enhance sensitivity and specificity of the search process. Truncation symbols, phrase searching, and database-specific indexing adaptations were additionally utilized where appropriate to improve retrieval accuracy. To minimize publication omission and improve comprehensiveness, backward reference list screening of included articles and relevant reviews was also undertaken manually to identify additional eligible studies that may not have been captured during electronic database searching. The full search strategy is summarized in Table 1. A detailed search strategy for each database is shown in Appendix A1–A5.

**Table 1:** Search strategy for the review.

Primary keywords	MeSH terms and free-text terms combined with Boolean operators	Boolean operators used	Databases
Convolutional neural networks	“Convolutional Neural Networks” OR “CNN” OR “Deep Convolutional Neural Network” OR “DCNN”	OR	PubMed, Scopus, Web of Science
Deep learning	“Deep Learning” OR “Artificial Intelligence” OR “Machine Learning”	OR	PubMed, IEEE Xplore
Medical imaging	“Medical Imaging” OR “Diagnostic Imaging” OR “Radiology” OR “Computer-Assisted Diagnosis”	OR	PubMed, ScienceDirect
Medical image analysis	“Image Interpretation, Computer-Assisted” OR “Image Analysis” OR “Medical Image Processing”	OR	PubMed, IEEE Xplore
Resource-constrained settings	“Resource-Limited Settings” OR “Low-Resource Settings” OR “Underserved Healthcare”	OR	Scopus, Web of Science
Low- and middle-income countries	“Developing Countries” OR “LMICs” OR “Low-Income Countries” OR “Middle-Income Countries”	OR	PubMed, Scopus
AI deployment	“AI Deployment” OR “Clinical Implementation” OR “Model Deployment”	OR	IEEE Xplore, ScienceDirect
Explainable AI	“Explainable Artificial Intelligence” OR “XAI” OR “Model Interpretability” OR “Grad-CAM”	OR	IEEE Xplore, Scopus
CNN architectures	“ResNet” OR “DenseNet” OR “EfficientNet” OR “MobileNet” OR “U-Net”	OR	IEEE Xplore, Web of Science
Diagnostic performance	“Accuracy” OR “Sensitivity” OR “Specificity” OR “AUC” OR “Precision”	OR	PubMed, Scopus
Offline AI systems	“Offline Inference” OR “Edge AI” OR “On-device AI”	OR	IEEE Xplore
Disease detection	“Disease Classification” OR “Lesion Detection” OR “Image Segmentation”	OR	PubMed, ScienceDirect
Clinical decision support	“Clinical Decision Support Systems” OR “Computer-Aided Diagnosis”	OR	PubMed
Deployment challenges	“Infrastructure Barriers” OR “Hardware Limitations” OR “Electricity Instability”	OR	Scopus, Web of Science
Ethical AI	“Algorithmic Bias” OR “Fairness” OR “Ethical AI” OR “Health Equity”	OR	Scopus, PubMed
Combined search example	(“Convolutional Neural Networks” OR “Deep Learning”) AND (“Medical Imaging” OR “Diagnostic Imaging”) AND (“Resource-Constrained Settings” OR “LMICs”)	AND/OR	All databases
Exclusion refinement	NOT (“Veterinary Imaging” OR “Non-medical Imaging”)	NOT	PubMed, Scopus

Comprehensive database search strategy showing keywords, MeSH terms, free-text combinations, and Boolean operators used for evidence retrieval.

### Eligibility criteria

The eligibility criteria for this scoping review were developed using the population–concept–context (PCC) framework recommended by the Joanna Briggs Institute for scoping reviews (Peters et al., 2020). Under the Population component, studies involving human medical imaging datasets, patients, healthcare workers, or healthcare systems utilizing CNN-based imaging technologies were considered eligible. Studies exclusively involving non-human, veterinary, or industrial imaging applications were excluded. Regarding the Concept component, studies were included if they explored convolutional neural networks or related deep learning architectures applied to medical imaging tasks such as classification, segmentation, object detection, disease diagnosis, or image interpretation. Studies focusing solely on traditional machine learning methods without CNN integration were excluded. For the Context component, studies conducted within resource-constrained, low-resource, underserved, or low- and middle-income healthcare settings were prioritized, including studies discussing implementation barriers relevant to such settings even if conducted in simulated environments. High-resource implementation studies lacking discussion of applicability to low-resource healthcare systems were excluded. Additional eligibility criteria included peer-reviewed original research articles published in English language journals. Conference abstracts without full-text availability, editorials, commentaries, protocols, dissertations, and non-peer-reviewed gray literature were excluded to ensure methodological robustness and evidence credibility.

### Study selection

All identified records retrieved from the database searches were exported into Zotero reference management software for citation organization and duplicate removal. Following de-duplication, the screened records were transferred into Microsoft Excel spreadsheets for systematic title, abstract, and full-text screening. Two reviewers independently screened titles, abstracts and full texts against the eligibility criteria. Any disagreements were resolved through discussion until consensus was achieved. Where consensus could not initially be reached, a third reviewer adjudicated. The study selection process was documented using the PRISMA-ScR flow diagram. Initially, titles and abstracts were screened against the predefined eligibility criteria, followed by full-text assessment of potentially relevant articles. Studies failing to satisfy the inclusion criteria were excluded at each stage with documented justification. To enhance transparency and reproducibility, the PRISMA-ScR flow diagram was used to record the identification, screening, eligibility assessment, inclusion process, and reasons for full-text article exclusion. The iterative screening process also enabled refinement of thematic relevance and ensured that only studies directly aligned with CNN-based medical imaging in resource-constrained settings were included in the final evidence synthesis.

### Data extraction

Data extraction was performed independently by two reviewers using a standardized Excel extraction form. Extracted information was cross-checked for consistency, and disagreements were resolved through discussion before final synthesis. The extraction template was piloted and refined iteratively to ensure consistency across included studies. Key study characteristics extracted included author(s), year of publication, country or geographical setting, study aim, study design, imaging modality, disease focus, dataset characteristics, sample size, CNN architecture utilized, computational platform, performance metrics (including accuracy, sensitivity, specificity, F1-score, and area under the curve), explainability approaches, deployment methods, implementation challenges, hardware requirements, offline capability, and key conclusions. Additional information relating to ethical considerations, bias mitigation strategies, external validation practices, and contextual applicability to resource-constrained healthcare environments was also extracted where available. This comprehensive extraction process enabled robust mapping of both technical and contextual evidence across the included studies.

### Critical appraisal

Consistent with JBI guidance for scoping reviews, formal methodological quality appraisal of included studies was not undertaken because the objective of this review was to comprehensively map the available evidence rather than determine intervention effectiveness or generate pooled estimates.

### Data synthesis

Data synthesis was conducted using a thematic descriptive approach consistent with scoping review methodology. Extracted findings were organized into major thematic domains relating to CNN architectures, diagnostic performance outcomes, explainability and interpretability approaches, deployment feasibility, infrastructure requirements, ethical considerations, and implementation barriers within resource-constrained healthcare settings. Studies were comparatively analyzed to identify recurring patterns, methodological inconsistencies, technological advancements, and evidence gaps across different imaging modalities and healthcare contexts. The synthesis additionally explored variations in model performance relative to dataset diversity, computational requirements, offline functionality, and clinical applicability in low-resource settings. A narrative analytical approach was employed to critically integrate findings while highlighting areas requiring further research, particularly regarding fairness, generalizability, external validation, and sustainable deployment of CNN systems in underserved healthcare environments.

## Results

### Overview of the search process

**Figure 1:**
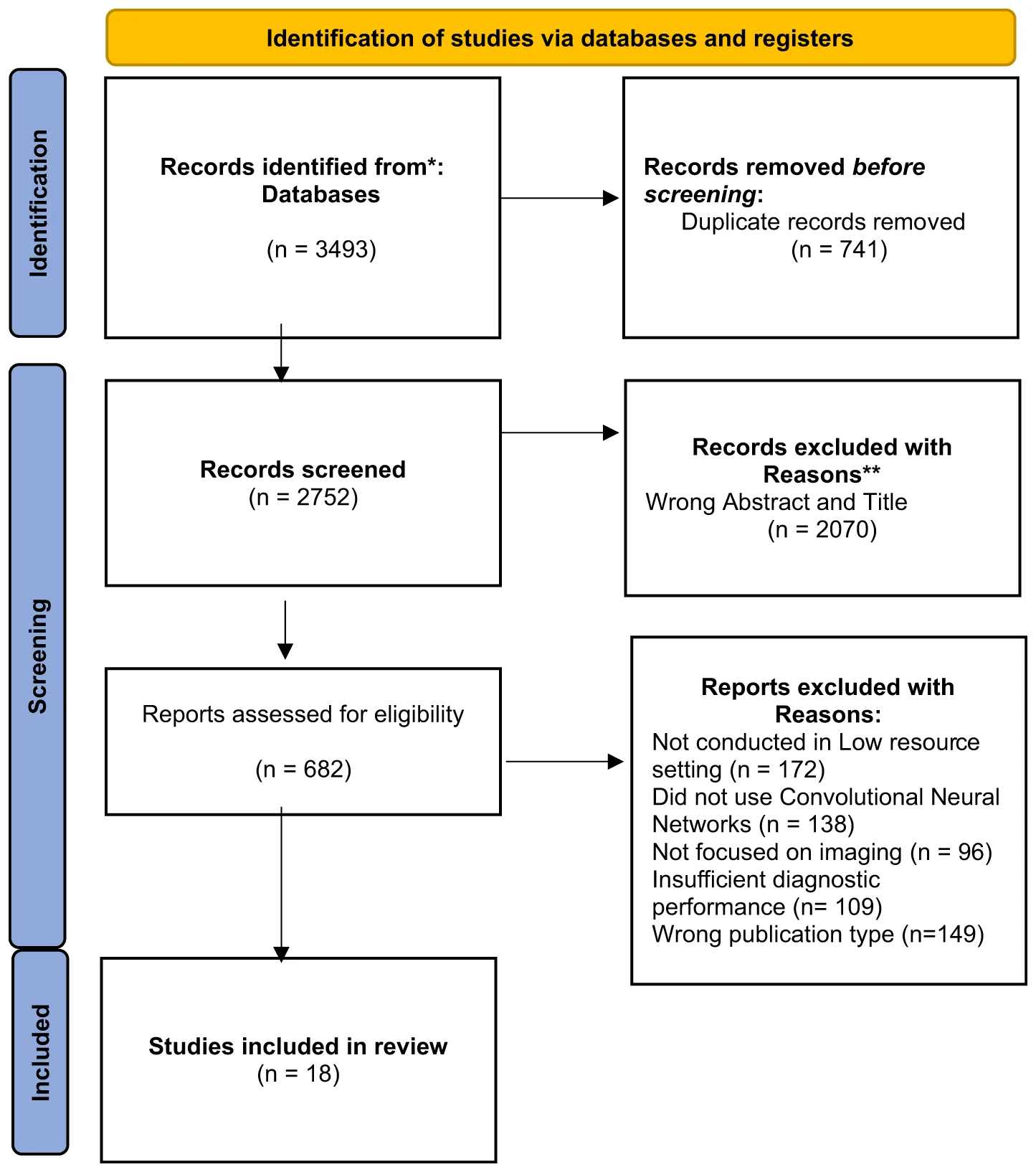
PRISMA flow diagram showing the study selection process (Adapted from Page et al., 2021). *Databases and registers searched for this review” and “**Automation tools and other criteria used to exclude records.

Following a comprehensive search across multiple electronic databases, a total of 3,493 records were initially identified for potential inclusion in this scoping review. After the removal of 741 duplicate records using Zotero reference management software, 2,752 studies remained for title and abstract screening. During the preliminary screening stage, 2,070 records were excluded due to irrelevance to the review topic, inappropriate study focus, or failure to meet the predefined eligibility criteria. Subsequently, 682 full-text articles were assessed for eligibility, of which 664 were excluded for reasons including unsuitable study settings, non-CNN methodologies, non-imaging focus, insufficient diagnostic reporting, and inappropriate publication types. Ultimately, 18 studies met the final inclusion criteria for this review. Although a large number of studies were initially retrieved, most were excluded because they did not specifically evaluate CNN applications within resource-constrained healthcare settings, focused on high-resource environments without implementation relevance, evaluated non-CNN algorithms, or lacked imaging-based diagnostic outcomes. The stringent eligibility criteria intentionally prioritized contextual relevance over study quantity. Figure 1 below presents the PRISMA flow diagram illustrating the complete study selection process.

### Characteristics of included studies

The 18 studies included in this scoping review demonstrated substantial diversity in study design, imaging modality, disease focus, convolutional neural network (CNN) architectures, and implementation settings. Most studies employed quantitative deep learning experimental designs using retrospective imaging datasets, with sample sizes ranging from several hundred to over 120,000 medical images. Chest X-ray imaging for tuberculosis, pneumonia, and COVID-19 detection represented the most frequently investigated modality, followed by microscopy-based malaria diagnostics, dermoscopic skin lesion analysis, histopathology, computed tomography (CT), and magnetic resonance imaging (MRI). Commonly utilized architectures included ResNet, DenseNet, MobileNet, VGGNet, EfficientNet, U-Net, and custom CNN models. Several studies additionally explored explainability, lightweight deployment, transfer learning, offline inference capability, and diagnostic applicability within resource-constrained healthcare environments. Detailed characteristics of the included studies are presented in Table 2.

**Table 2:** Characteristics of included studies.

Studies	Country/setting	Study design	Disease/clinical focus	Imaging modality	Sample size/dataset	CNN architecture/model	Key performance findings	Resource-constrained/deployment relevance	Key findings/conclusions
Esteva et al. (2017)	USA	Quantitative experimental study	Skin cancer classification	Dermoscopic clinical images	129,450 skin lesion images	GoogleNet/Inception v3 CNN	AUC of 0.96; dermatologist-level performance	Potential smartphone-based dermatology screening	CNN achieved expert-level classification accuracy for skin cancer diagnosis
Quinn et al. (2016)	USA	Retrospective quantitative study	Pulmonary tuberculosis detection	Chest X-ray	1,007 chest radiographs	AlexNet and GoogLeNet	Accuracy 96%; AUC 0.99	Useful for automated TB screening in low-resource settings	CNN models effectively classified TB from chest radiographs
Rajpurkar et al. (2017)	USA	Quantitative deep learning study	Pneumonia detection	Chest X-ray	112,120 X-ray images	CheXNet (121-layer DenseNet)	F1-score exceeded radiologists in pneumonia detection	Supports AI-assisted radiology shortage management	Deep CNN showed radiologist-level pneumonia detection
Quinn et al. (2016)	Uganda	Experimental quantitative study	Malaria parasite detection	Microscopy images	Thousands of blood smear images	Custom deep CNN	High sensitivity and specificity	Point-of-care microscopy diagnostics	CNNs demonstrated feasibility for low-cost malaria diagnostics
Rajaraman et al. (2018a)	Thailand/Bangladesh	Quantitative comparative study	Malaria parasite identification	Thin blood smear microscopy	27,558 cell images	Pre-trained AlexNet, VGG-16, Xception	Accuracy above 95%	Supports automated microscopy interpretation	Transfer learning improved malaria parasite detection
Rajaraman et al. (2018b)	Thailand	Quantitative experimental study	Malaria parasite detection	Thin blood smear microscopy	27,558 cell images	Customized CNN models	Improved explainability and classification reliability	Relevant for explainable AI diagnostics in LMICs	CNN visualization improved interpretability of parasite detection
Liu et al. (2023)	Taiwan	Cross-sectional study	Tuberculosis and NTM-LD detection	Chest X-ray	6,037 chest X-rays	Deep learning CNN model	AUC >0.91	Potential low-resource TB triage tool	CNN effectively differentiated TB and NTM-LD
Sharma et al. (2024)	India	Quantitative imaging study	Tuberculosis detection	Chest X-ray	Public TB datasets	ResNet50, EfficientNet	Accuracy >95%	Visualization supports explainability in low-resource care	Deep learning models improved TB localization and diagnosis
Sarawagi et al. (2024)	India	Quantitative self-training study	Tuberculosis detection	Chest X-ray	Multi-source chest X-ray datasets	Self-trained CNN	Improved sensitivity and specificity	Semi-supervised learning useful where labeled data are limited	Self-training CNN improved TB screening performance
Tajbakhsh et al. (2016)	USA	Comparative quantitative study	General medical imaging analysis	Multiple imaging modalities	Various benchmark medical datasets	Fine-tuned CNN architectures	Fine-tuning outperformed full training in limited datasets	Relevant for small-resource healthcare datasets	Transfer learning beneficial for medical image analysis
Spanhol et al. (2016)	Brazil	Quantitative classification study	Breast cancer histopathology	Histopathological images	7,909 microscopic images	CNN classifier	Accuracy >85%	Automated pathology interpretation potential	CNN accurately classified histopathological breast cancer images
Varshni et al. (2019)	India	Quantitative experimental study	Pneumonia detection	Chest X-ray	Pediatric chest X-ray dataset	CNN feature extraction model	Accuracy approximately 93%	Useful for low-cost pneumonia screening	CNN-based feature extraction improved pneumonia diagnosis
Maqsood et al. (2019)	Pakistan	Quantitative deep learning study	Alzheimer's disease staging	MRI	ADNI MRI datasets	Transfer learning CNN	Accuracy >98%	Demonstrates low-data optimization potential	Transfer learning improved MRI-based Alzheimer staging
Hua et al. (2015)	Taiwan	Quantitative imaging analysis	Lung nodule classification	CT imaging	Lung CT image datasets	Deep CNN	Accuracy approximately 84%	Computer-aided diagnosis support	Deep learning improved lung nodule classification
Sedik et al. (2020)	Egypt/Saudi Arabia	Quantitative comparative study	COVID-19 detection	Chest X-ray and CT	Multi-source COVID imaging datasets	CNN and machine learning hybrids	Accuracy >94%	Rapid screening applicability in overwhelmed systems	Deep learning enabled efficient COVID-19 detection
Khan et al. (2020)	India	Quantitative diagnostic study	COVID-19 diagnosis	Chest X-ray	Public COVID chest X-ray datasets	CoroNet CNN	Accuracy 89.6%	Lightweight architecture for rapid deployment	CNN demonstrated rapid COVID-19 screening capability
Owda et al. (2025)	Saudi Arabia	Quantitative lightweight AI study	Tuberculosis detection	Chest X-ray	Large public TB datasets	Lightweight hybrid CNN	High sensitivity with lower computational demand	Designed for low-resource deployment environments	Lightweight models improved deployment feasibility
Mirugwe et al. (2025)	Malawi/Uganda	Comparative quantitative study	Tuberculosis screening	Chest X-ray	Multiple TB chest X-ray datasets	ResNet, DenseNet, EfficientNet	EfficientNet achieved superior balanced performance	Transfer learning beneficial for African healthcare settings	Comparative CNN architectures improved TB detection performance

### Findings

Analysis of the 18 included studies revealed five major recurring themes relating to the application of CNNs in medical imaging within resource-constrained healthcare environments. These themes reflected the dominant research priorities across the literature and included: (1) diagnostic accuracy and clinical performance of CNN models, (2) lightweight and resource-efficient CNN architectures for low-resource deployment, (3) explainability and interpretability of AI systems, (4) transfer learning and optimization for limited datasets, and (5) implementation barriers and infrastructural deployment challenges. Collectively, these themes demonstrated both the growing potential and operational limitations of CNN-driven medical imaging systems in underserved healthcare settings.

## Theme 1: diagnostic accuracy and clinical performance of CNN models

Most included studies primarily evaluated the diagnostic performance of CNN-based systems across multiple imaging modalities and disease conditions (Table 3). Within clinical diagnostics, measures such as sensitivity, specificity, accuracy, F1-score, and area under the receiver operating characteristic curve (AUC) are frequently used to evaluate the discriminatory power and reliability of AI-based imaging systems. Across the included studies, CNN models consistently demonstrated strong diagnostic performance across several imaging modalities, including chest radiography, dermoscopy, microscopy, histopathology, computed tomography (CT), and magnetic resonance imaging (MRI). However, although many studies reported high predictive performance, the methodological rigor, dataset representativeness, and real-world applicability varied substantially between studies, particularly in relation to deployment within resource-constrained healthcare environments.

**Table 3:** CNN diagnostic performance across included studies.

Study	Disease focus	Imaging modality	CNN architecture	Key performance outcome
Esteva et al. (2017)	Skin cancer	Dermoscopy	Inception v3	AUC = 0.96
(Lakhani and Sundaram 2017)	Tuberculosis	Chest X-ray	AlexNet/GoogLeNet	Accuracy = 96%
Rajpurkar et al. (2017)	Pneumonia	Chest X-ray	DenseNet-121	Radiologist-level F1-score
Quinn et al. (2016)	Malaria	Microscopy	Custom CNN	High sensitivity/specificity
Sharma et al. (2024)	Tuberculosis	Chest X-ray	ResNet50/EfficientNet	Accuracy >95%
Sedik et al. (2020)	COVID-19	Chest X-ray/CT	CNN hybrids	Accuracy >94%

A substantial proportion of the included studies focused on chest X-ray interpretation for infectious disease diagnosis, particularly tuberculosis, pneumonia, and COVID-19 detection. Lakhani and Sundaram (2017) demonstrated that CNN architectures including AlexNet and GoogLeNet achieved diagnostic accuracies approaching 96% for pulmonary tuberculosis classification using chest radiographs, with AUC values nearing 0.99. These findings were particularly significant because tuberculosis remains one of the leading infectious causes of mortality globally, disproportionately affecting low- and middle-income countries where radiologist shortages remain severe. Similarly, Rajpurkar et al. (2017) developed the CheXNet model using a 121-layer DenseNet architecture trained on over 112,000 chest X-rays, reporting pneumonia detection performance comparable to practicing radiologists. The scale of the dataset used in this study substantially strengthened the internal validity of the findings, although concerns remain regarding external generalizability to low-resource healthcare settings where imaging quality, patient demographics, and disease presentations may differ significantly from the original training environment. Comparable findings were reported by Sharma et al. (2024), who applied ResNet50 and EfficientNet architectures for tuberculosis detection and infected region localization, achieving diagnostic accuracies exceeding 95%. Importantly, this study integrated visual localization approaches that improved interpretability of disease prediction, suggesting that explainability mechanisms may enhance clinical confidence in AI-supported diagnosis.

The application of CNNs in microscopy-based diagnostics also emerged prominently within the included evidence, particularly for malaria parasite detection. Quinn et al. (2016) explored deep CNNs for point-of-care microscopy diagnostics and demonstrated that automated parasite recognition could achieve high sensitivity and specificity despite variations in smear quality and imaging conditions. This finding is particularly relevant in resource-constrained settings where access to trained microscopists is limited and diagnostic inconsistency contributes substantially to delayed treatment initiation. Rajaraman et al. (2018a) similarly evaluated pre-trained CNN architectures including AlexNet, VGG-16, and Xception for malaria parasite identification using thin blood smear images and reported classification accuracies exceeding 95%. The study further demonstrated that transfer learning approaches substantially improved feature extraction efficiency and classification performance in relatively small datasets. Nevertheless, although the reported performance metrics were promising, both studies highlighted an important limitation regarding dataset heterogeneity and external validation. Most imaging datasets were derived from controlled laboratory settings with relatively standardized image acquisition conditions, thereby raising concerns regarding transferability to rural diagnostic environments characterized by inconsistent staining procedures, low-resolution microscopes, and variable lighting conditions.

Beyond infectious disease imaging, several studies demonstrated strong CNN performance across broader diagnostic domains including dermatology, oncology, and neuroimaging. Esteva et al. (2017) reported dermatologist-level skin cancer classification using the Inception v3 CNN architecture trained on over 129,000 dermoscopic images. The study achieved AUC values of approximately 0.96 and represented one of the earliest large-scale demonstrations of expert-level CNN diagnostic capability in dermatological imaging. Similarly, Spanhol et al. (2016) showed that CNN-based histopathological image classification achieved accuracies exceeding 85% for breast cancer diagnosis, while Hua et al. (2015) demonstrated effective lung nodule classification from CT images using deep CNN models. Maqsood et al. (2019) further extended CNN application into MRI-based Alzheimer's disease staging, reporting accuracies above 98% through transfer learning optimization. Despite these promising findings, the studies collectively exposed a recurring methodological tension between algorithmic performance under experimental conditions and real-world clinical integration. Several models achieved high accuracy primarily because they were trained on curated public datasets with minimal image artifacts and balanced disease distributions, conditions rarely replicated in low-resource healthcare environments. Consequently, while the evidence strongly supports the diagnostic potential of CNNs across multiple imaging domains, the literature simultaneously reveals persistent uncertainty regarding the reproducibility, robustness, and operational validity of these systems in routine frontline healthcare delivery within underserved settings.

## Theme 2: lightweight and resource-efficient CNN architectures for low-resource deployment

In many LMICs, healthcare systems frequently operate under substantial infrastructural constraints including limited GPUs, low-memory computing devices, unstable electricity supply, and inconsistent internet connectivity. Consequently, several studies within this review prioritized the development of lightweight and resource-efficient CNN architectures capable of maintaining acceptable diagnostic performance while reducing computational burden. This shift reflects a broader recognition that highly complex deep learning systems with extensive parameter sizes may demonstrate strong performance in laboratory conditions yet remain impractical for real-world implementation in underserved healthcare facilities (Table 4).

**Table 4:** Lightweight CNN architectures and deployment relevance.

Study	Lightweight/optimized architecture	Resource-constrained relevance	Key deployment benefit
Khan et al. (2020)	CoroNet CNN	Rapid low-resource COVID screening	Faster inference
Owda et al. (2025)	Lightweight hybrid CNN	Reduced hardware requirements	Lower computational cost
Sharma et al. (2024)	EfficientNet	Explainable TB diagnosis	Efficient image processing
Mirugwe et al. (2025)	EfficientNet/DenseNet	African healthcare applicability	Balanced performance
Tajbakhsh et al. (2016)	Fine-tuned CNNs	Small dataset optimization	Reduced training burden

Khan et al. (2020) addressed this challenge through the development of CoroNet, a lightweight CNN architecture designed for rapid COVID-19 detection from chest X-ray images. The model was intentionally optimized to reduce computational demand while preserving clinically relevant diagnostic performance, achieving approximately 89.6% accuracy despite operating with substantially lower processing requirements than larger conventional CNN architectures. The significance of this approach lies not solely in diagnostic accuracy, but in the model's potential adaptability to low-resource clinical environments where high-performance computational infrastructure may be unavailable. Importantly, the study demonstrated that simplified architectures could still produce meaningful diagnostic outcomes without requiring extensive cloud-based processing systems. However, although the architecture was computationally efficient, the study remained dependent on retrospectively curated public datasets, thereby limiting understanding of how such lightweight systems would perform under variable real-world imaging conditions commonly observed in rural healthcare facilities.

Similarly, Owda et al. (2025) developed a lightweight hybrid deep learning framework for tuberculosis detection using chest radiographs, explicitly focusing on computational optimization for low-resource deployment. The study reported that reducing architectural complexity and parameter density substantially improved inference speed and hardware compatibility while maintaining high sensitivity for tuberculosis classification. Unlike earlier high-capacity CNN systems, the lightweight hybrid model demonstrated greater feasibility for integration into portable diagnostic platforms and edge-based AI systems. This finding is particularly important because tuberculosis screening in many LMICs occurs in geographically isolated settings where internet-dependent AI infrastructures are difficult to sustain. The study therefore reinforced the argument that deployment feasibility should be considered alongside predictive accuracy when evaluating CNN suitability for resource-constrained healthcare systems. Nevertheless, despite the promising computational efficiency observed, the study provided limited evaluation regarding long-term clinical integration, maintenance costs, and interoperability with existing healthcare information systems, which remain important determinants of sustainability in underserved settings.

Resource-efficient CNN deployment was also closely associated with the increasing preference for mobile-compatible and edge-based AI systems capable of offline inference. Mirugwe et al. (2025) compared multiple CNN architectures including ResNet, DenseNet, and EfficientNet for tuberculosis screening in African healthcare contexts and reported that EfficientNet achieved a more favorable balance between computational efficiency and diagnostic performance. EfficientNet's compound scaling approach enabled optimization of model depth, width, and resolution simultaneously, reducing unnecessary computational expansion while preserving classification accuracy. Sharma et al. (2024) similarly demonstrated that EfficientNet-based architectures provided effective tuberculosis localization and explainability with reduced computational burden compared to heavier CNN frameworks. These findings collectively suggest that efficient CNN architectures may offer more realistic implementation pathways for AI-assisted diagnostics in healthcare systems characterized by infrastructure limitations and workforce shortages. Nonetheless, the literature simultaneously reveals that many studies continue to evaluate deployment readiness under controlled experimental conditions rather than real-world clinical environments where device variability, unstable power supply, image quality inconsistency, and limited technical support may substantially affect model functionality and scalability.

## Theme 3: explainability and interpretability of CNN systems

In healthcare environments, diagnostic systems are expected not only to provide accurate predictions but also to demonstrate transparency and accountability, especially when clinical decisions may directly affect patient outcomes. Across the included studies, explainability emerged as a critical determinant influencing clinician trust, adoption feasibility, and practical integration of CNN-based diagnostic tools. This concern becomes even more significant within resource-constrained settings where specialist radiologists, pathologists, or laboratory scientists may be limited, thereby increasing reliance on AI-generated diagnostic outputs without immediate expert verification.

Rajaraman et al. (2018b) provided one of the clearest demonstrations of explainable CNN implementation within microscopy-based malaria diagnostics. The study investigated customized CNN architectures for malaria parasite detection and incorporated visualization techniques to understand the learned behavior of the models during classification. Through activation mapping and feature visualization strategies, the authors demonstrated that the CNN models focused attention on biologically relevant parasite structures rather than unrelated image artifacts. This finding was particularly important because it addressed a recurring criticism of deep learning systems: the possibility that models may generate high accuracy by learning irrelevant background correlations instead of clinically meaningful pathological features. By improving visibility into feature extraction processes, the study strengthened confidence in the biological plausibility of CNN predictions. Nevertheless, the study also exposed a broader methodological challenge, namely that explainability methods remain largely interpretive rather than fully causal, meaning that highlighted regions do not necessarily prove clinically valid reasoning pathways within the model architecture itself.

Similar concerns regarding interpretability were evident in Sharma et al. (2024), who integrated localization methods and visual heatmaps into tuberculosis detection systems using chest X-ray imaging. Their study demonstrated that explainability mechanisms could visually identify infected pulmonary regions contributing to CNN-based predictions, thereby improving diagnostic transparency and supporting clinician verification of automated outputs. Importantly, this approach may be particularly beneficial in low-resource healthcare systems where frontline healthcare workers with limited radiological training rely on AI-assisted decision support for rapid triage and screening. The integration of visual explanation tools therefore extends beyond technical functionality and enters the domain of clinical communication, enabling healthcare professionals to understand why particular predictions were generated. However, despite these advantages, Sharma et al. (2024) acknowledged that explainability tools may still oversimplify complex neural representations and occasionally produce unstable or inconsistent localization outputs depending on image quality and model architecture. Consequently, although heatmaps and attention maps improve interpretability, they do not entirely eliminate concerns regarding diagnostic reliability or hidden model biases.

The broader literature included within this review further demonstrated that explainability intersects closely with ethical accountability and clinician acceptance of AI technologies. Quinn et al. (2016), while primarily focusing on microscopy-based point-of-care diagnostics, highlighted that healthcare workers were more likely to trust automated systems when outputs aligned visibly with identifiable pathological regions. This reflects a fundamental distinction between predictive accuracy and clinical credibility. A model may achieve statistically high performance metrics yet still face resistance if healthcare workers cannot interpret the reasoning behind predictions. Similarly, Esteva et al. (2017) demonstrated dermatologist-level skin cancer classification using CNN architectures but simultaneously raised concerns regarding the interpretability of highly complex deep learning models trained on massive image repositories. The study illustrated that although deep CNNs may outperform traditional diagnostic methods in predictive capability, increasing model complexity often reduces transparency, thereby complicating clinician oversight and medico-legal accountability. This tension between predictive sophistication and interpretability remains a central challenge across medical AI research.

Explainability also emerged as particularly important in resource-constrained healthcare settings because diagnostic uncertainty may carry greater consequences where confirmatory testing and specialist consultation are limited. Studies included within this review collectively suggested that interpretable CNN systems may improve diagnostic confidence among frontline healthcare workers, facilitate safer triage decisions, and reduce reluctance toward AI adoption. Nonetheless, the literature simultaneously revealed that many explainability techniques remain experimentally oriented and insufficiently validated in real-world clinical environments. Several models demonstrated strong visual explanation capability under curated datasets yet lacked evaluation under poor-quality imaging conditions frequently encountered in rural healthcare facilities. Moreover, while explainability tools such as Grad-CAM, saliency mapping, and activation heatmaps provide visual insight into model attention, they do not inherently guarantee fairness, reliability, or absence of algorithmic bias. Consequently, the evidence indicates that explainability should not be viewed merely as an auxiliary feature of CNN systems, but rather as a foundational requirement for ethically acceptable and clinically sustainable deployment of AI-assisted medical imaging within underserved healthcare systems.

## Theme 4: transfer learning and optimization for limited datasets

Transfer learning refers to a deep learning strategy in which knowledge acquired by a neural network during training on a large source dataset is transferred to a related target task, thereby reducing the amount of labeled data, computational resources, and training time required for model development (Rajaraman et al., 2018a). Rather than learning image features from randomly initialized parameters, pre-trained CNNs utilize previously learned low-level and high-level feature representations, which can subsequently be fine-tuned for disease-specific medical imaging applications. This approach has become particularly valuable in resource-constrained healthcare settings, where limited access to large annotated datasets, specialist expertise, and high-performance computing infrastructure frequently restricts the development of robust deep learning models (Esteva et al., 2017).

One of the most persistent challenges in medical imaging research within resource-constrained environments relates to the availability of sufficiently large and well-annotated datasets required for effective deep learning model development. CNNs generally require extensive training datasets to learn hierarchical image features and optimize predictive performance. However, generating large medical imaging repositories is often difficult in low- and middle-income countries (LMICs) due to limitations in digital infrastructure, specialist expertise, financial resources, and standardized data collection systems. Manual annotation of medical images frequently requires experienced clinicians, radiologists, or laboratory scientists, which may substantially increase costs and time requirements. Consequently, transfer learning emerged across the included studies as a practical optimization strategy aimed at addressing data scarcity while simultaneously reducing computational demand and training complexity.

Transfer learning refers to the process through which a neural network model trained on one large dataset applies previously acquired knowledge to a different but related task. Rather than constructing models from the beginning using randomly initialized parameters, pre-trained CNN architectures utilize existing feature representations developed from prior learning processes, thereby accelerating model convergence and reducing the quantity of labeled data required for effective classification. Within medical imaging applications, transfer learning commonly employs established architectures such as VGGNet, AlexNet, ResNet, DenseNet, or Inception networks that have initially been trained on large-scale datasets before adaptation to disease-specific imaging tasks. Across the included studies, evidence consistently demonstrated that transfer learning approaches significantly improved classification outcomes while reducing computational burden, making them particularly relevant for resource-limited healthcare systems.

Tajbakhsh et al. (2016) conducted one of the earliest comparative investigations examining whether full CNN training or fine-tuning of pre-trained architectures produced superior medical imaging performance. Their findings demonstrated that fine-tuned CNN models consistently outperformed models trained entirely from scratch across multiple imaging applications, particularly when smaller datasets were available. The study further showed that transfer learning substantially reduced training time and computational requirements without compromising predictive performance. These findings possess important implications for LMIC healthcare settings because generating large disease-specific image repositories remains a major logistical challenge. Rather than requiring extensive computing infrastructure and large annotated datasets, fine-tuning strategies allow healthcare researchers to maximize existing resources while achieving clinically meaningful diagnostic performance. Nevertheless, despite these advantages, the study also identified that transfer learning effectiveness varies depending on similarity between source and target domains, suggesting that features learned from unrelated datasets may occasionally provide limited diagnostic benefit.

Comparable findings were observed in Rajaraman et al. (2018a), who explored pre-trained CNN models including AlexNet, VGG-16, and Xception for malaria parasite detection using thin blood smear microscopy images. The study demonstrated that transfer learning approaches achieved classification accuracies exceeding 95%, substantially improving feature extraction efficiency compared to conventional training methods. The authors argued that reusing previously learned image representations allowed the model to identify subtle morphological parasite characteristics despite limited dataset availability. This finding is particularly relevant because malaria diagnostics in many resource-constrained settings frequently rely on microscopy-based approaches where digital image datasets may be scarce and heterogeneous. However, although transfer learning improved classification outcomes, the study simultaneously revealed concerns regarding dataset dependence, as image acquisition conditions remained relatively controlled and standardized. Consequently, uncertainties remain regarding whether such optimized models would retain comparable performance under routine field conditions characterized by poor image quality, inconsistent staining procedures, and variable microscope specifications.

Further evidence supporting transfer learning optimization was reported by Maqsood et al. (2019), who investigated MRI-based Alzheimer's disease staging using pre-trained CNN models integrated with transfer learning techniques. The study achieved diagnostic accuracies exceeding 98%, demonstrating that previously acquired feature representations could substantially improve classification performance even within specialized neuroimaging contexts. Importantly, the authors observed considerable reductions in computational complexity and training duration compared with conventional deep learning approaches. Although Alzheimer's disease imaging differs substantially from infectious disease diagnostics commonly encountered in resource-constrained settings, the methodological implications remain highly relevant because the study illustrates how transfer learning may compensate for limitations in data quantity and computational resources. However, an important concern emerging across the evidence relates to the origin of pre-trained datasets themselves. Most foundational CNN architectures have been trained predominantly on large image repositories derived from high-income populations and non-medical imaging collections, raising questions regarding feature transferability across diverse demographic and disease contexts. Consequently, while transfer learning offers considerable potential for optimizing CNN deployment in resource-constrained healthcare systems, the literature also suggests that overreliance on externally trained models may inadvertently introduce bias, reduce contextual relevance, and limit broader generalizability within underserved populations.

## Theme 5: implementation barriers and deployment challenges in resource-constrained settings

The distinction between experimental effectiveness and operational feasibility emerged repeatedly across the literature, particularly within low-resource environments where infrastructural limitations frequently constrain technology adoption. Resource-constrained settings are typically characterized by deficiencies in computing infrastructure, specialist personnel shortages, unreliable electricity supply, inadequate digital systems, and restricted financial resources, all of which may significantly influence the functionality and sustainability of artificial intelligence (AI)-assisted medical imaging systems. Consequently, implementation barriers appeared not merely as technical concerns but as complex healthcare systems challenges involving interactions between technology, infrastructure, workforce capacity, and contextual healthcare realities.

One of the most frequently identified barriers across the included studies related to limitations in computational infrastructure required for CNN deployment. Deep learning models generally require considerable processing capability, memory allocation, and graphical processing unit (GPU) support to perform image analysis effectively. However, such infrastructure remains scarce in many low- and middle-income healthcare systems. Quinn et al. (2016), in their investigation of microscopy-based point-of-care diagnostics, observed that although CNN models achieved promising diagnostic performance, implementation within rural healthcare environments required substantial adaptation due to hardware limitations and restricted access to high-performance computational systems. Their findings suggested that dependence on computationally intensive architectures may reduce practicality in healthcare facilities where diagnostic equipment itself may be limited. Similarly, Owda et al. (2025) highlighted that reducing model complexity and computational requirements was necessary to facilitate deployment in environments characterized by constrained processing resources. Nevertheless, while lightweight approaches improved computational efficiency, the study acknowledged that optimization strategies alone cannot compensate for broader infrastructural deficits such as insufficient hardware maintenance systems and limited technical expertise among healthcare personnel.

Internet dependence and unstable electricity supply also emerged as significant operational constraints affecting deployment feasibility across resource-constrained healthcare settings. AI-assisted diagnostic systems frequently rely on cloud-based computational infrastructures for model processing, storage, and updating mechanisms. However, healthcare facilities within rural or underserved regions commonly experience unreliable internet access and frequent power interruptions that may substantially disrupt AI-supported workflows. Mirugwe et al. (2025) demonstrated that even highly optimized CNN architectures designed for tuberculosis detection could face implementation challenges if deployment models relied excessively on internet-enabled processing environments. Their comparative investigation of multiple CNN architectures highlighted the importance of locally adaptable and offline-capable systems capable of functioning independently of continuous network connectivity. These findings are particularly relevant because healthcare delivery in many underserved regions frequently occurs in geographically isolated areas where stable communication infrastructure remains underdeveloped. Consequently, dependence on external computational support systems may inadvertently widen healthcare inequalities rather than reduce them if technological solutions are designed primarily for well-resourced environments.

Another recurring challenge involved limited availability of annotated medical imaging datasets required for CNN development and validation. Deep learning algorithms depend heavily on high-quality labeled data for effective feature extraction and predictive optimization. However, image annotation frequently requires expert clinical interpretation, creating substantial logistical and financial demands that many healthcare systems struggle to meet. Quinn et al. (2016) noted that obtaining adequately labeled microscopy datasets for malaria diagnostics represented a considerable challenge, particularly in settings where specialist microscopists were scarce. Similarly, Mirugwe et al. (2025) identified limitations relating to locally representative chest X-ray datasets for tuberculosis classification within African populations. These findings raise important concerns regarding transferability because models trained predominantly using datasets derived from high-income settings may inadequately capture disease variability, imaging characteristics, or demographic diversity observed within LMIC populations. Consequently, insufficient dataset representativeness may compromise diagnostic robustness and reduce external validity when models are introduced into diverse clinical environments.

Algorithmic bias also emerged as an important implementation challenge across the included evidence. Bias in AI systems may occur when training datasets inadequately represent particular demographic groups, healthcare settings, or disease characteristics, resulting in unequal predictive performance across populations. Although Owda et al. (2025) and Mirugwe et al. (2025) demonstrated encouraging diagnostic performance for tuberculosis detection systems, both studies acknowledged concerns regarding model generalizability beyond the original training populations. This issue becomes particularly significant within resource-constrained settings because underrepresentation of LMIC populations in CNN training datasets may increase risks of inaccurate classification and unequal healthcare outcomes. Furthermore, the literature repeatedly suggested that validation studies remain heavily concentrated within controlled research environments rather than real-world healthcare systems. Consequently, while the included studies collectively support the considerable diagnostic promise of CNN systems, they simultaneously reveal persistent uncertainty regarding operational readiness, sustainability, and contextual adaptability, suggesting that implementation challenges may ultimately represent a greater obstacle to healthcare impact than algorithmic performance itself.

## Discussion

This scoping review aimed to critically map the evidence regarding convolutional neural networks (CNNs) for medical imaging in resource-constrained settings, with emphasis on CNN architectures, diagnostic performance, and implementation challenges influencing sustainable deployment. The findings of this review identified five interconnected themes involving diagnostic accuracy and clinical performance, lightweight architectures for low-resource deployment, explainability and interpretability, transfer learning for limited datasets, and implementation barriers affecting real-world application. Comparison of these findings with broader literature suggests substantial agreement regarding the transformative potential of CNNs in medical imaging; however, important discrepancies remain regarding model generalizability, contextual deployment, and practical integration into healthcare systems.

The high diagnostic performance identified across the included studies aligns strongly with previous systematic and narrative reviews conducted across broader medical imaging contexts. Zhou et al. (2021) reported that CNN systems have consistently achieved state-of-the-art performance across several diagnostic tasks including disease classification, segmentation, lesion localization, and image enhancement, supporting the findings of the present review regarding strong performance in tuberculosis, pneumonia, malaria, and skin cancer detection. Similarly, Mienye et al. (2025) observed that CNN architectures such as ResNet, DenseNet, U-Net, and EfficientNet have substantially improved predictive capability across oncology, neurology, pulmonology, and dermatology applications. These findings collectively reinforce evidence suggesting that CNN models frequently approach or even exceed expert-level diagnostic performance under controlled experimental conditions. However, broader evidence simultaneously highlights concerns regarding overreliance on performance metrics such as accuracy and AUC as indicators of clinical readiness. Egger et al. (2022), in a systematic meta-review of medical deep learning literature, argued that reported performance measures frequently conceal substantial heterogeneity in dataset quality, external validation procedures, and population representativeness. This observation supports concerns emerging from the present review regarding transferability of high-performing CNN systems into underserved healthcare environments where patient populations and imaging conditions differ considerably from training datasets.

The findings relating to lightweight and computationally efficient architectures also demonstrate substantial consistency with wider evidence. This review found that models designed for reduced computational demand may improve deployment feasibility in low-resource settings where high-performance computational systems are unavailable. Similar observations were reported by Sistaninejhad et al. (2023), who noted that although CNN architectures have significantly improved image processing performance, computational complexity remains a major barrier limiting broader clinical adoption. Furthermore, Zhou et al. (2021) highlighted that resource-efficient network design and hardware optimization represent important emerging priorities in medical imaging research. Nevertheless, while broader literature recognizes the potential advantages of lightweight architectures, there remains uncertainty regarding whether reductions in computational burden may inadvertently compromise feature extraction capacity and predictive robustness. Several reviews suggest that although architectures such as MobileNet and EfficientNet demonstrate favorable efficiency-to-performance ratios, comparative evidence regarding long-term clinical effectiveness remains insufficient (Mienye et al., 2025). Consequently, there remains an unresolved methodological challenge concerning the balance between computational efficiency and preservation of clinically meaningful performance outcomes.

The importance of explainability identified within this review is also strongly supported by wider literature examining XAI in medical imaging systems. Liu et al. (2025) reported that clinician acceptance of AI systems depends substantially on the ability of models to provide meaningful and understandable explanations for diagnostic outputs. Their systematic review found that saliency mapping, Grad-CAM visualization, and local explanation approaches represented the most commonly utilized explainability methods in CNN-based imaging applications. Similarly, Zhou et al. (2021) highlighted interpretability as one of the most critical unresolved challenges limiting AI translation into clinical practice. While the present review found that explainability may strengthen diagnostic confidence among healthcare workers, broader literature suggests that current explanation techniques frequently provide descriptive rather than causal interpretations of CNN behavior. Consequently, visual explanation methods may improve perceived transparency without necessarily ensuring that diagnostic reasoning processes remain clinically valid. This distinction is important because explainability should not merely function as an interface feature designed to increase trust but should also provide evidence of underlying model reliability and accountability.

The present review additionally identified transfer learning as a practical strategy for addressing limitations in labeled datasets within resource-constrained healthcare systems. Similar findings have been reported extensively across previous reviews. Peng and Wang (2021) noted that limited supervision and inadequate annotation availability remain major challenges in medical image analysis and that transfer learning approaches frequently compensate for restricted dataset sizes through reuse of previously learned features. Likewise, Zhou et al. (2021) argued that transfer learning reduces computational demand while accelerating model convergence and improving predictive performance in small datasets. However, broader literature also identifies important limitations associated with this approach. Several reviews suggest that pre-trained architectures frequently originate from high-income populations or non-medical image datasets, potentially introducing biases and reducing contextual relevance when applied within underrepresented populations (Zhou et al., 2021; Egger et al., 2022). This concern directly reflects findings from the current review, which indicated uncertainties regarding the external validity of CNN models in resource-constrained settings.

Implementation barriers identified within this review similarly demonstrate considerable agreement with previous literature regarding challenges affecting real-world AI deployment. The World Health Organization has repeatedly highlighted diagnostic infrastructure limitations, workforce shortages, and digital health inequalities as major barriers affecting healthcare technology implementation globally (World Health Organization, 2023). Broader reviews examining medical AI deployment have similarly emphasized issues including insufficient computational infrastructure, internet dependency, data heterogeneity, algorithmic bias, and limited external validation procedures (Zhou et al., 2021; Mienye et al., 2025). However, while previous reviews generally discuss implementation barriers from a global perspective, the present review extends this evidence by highlighting the disproportionate influence of such challenges within underserved healthcare environments. Existing literature frequently assumes availability of stable technological infrastructure and specialist expertise, assumptions that may not accurately reflect realities within many LMIC healthcare systems. Consequently, although CNN models continue to demonstrate considerable diagnostic promise, the broader evidence increasingly suggests that future research priorities should move beyond performance optimization alone and place greater emphasis on contextual adaptability, local validation, and healthcare system integration.

Although recent advances including Vision Transformers, ConvNeXt, Segment Anything Models (SAM), MedSAM and multimodal foundation models are increasingly being investigated in medical imaging, these approaches were outside the scope of the present review, which specifically focused on convolutional neural network-based architectures. Table 5 shows the comparison of the present review with previous reviews on artificial intelligence and medical imaging. Future scoping reviews should comparatively evaluate these emerging AI paradigms alongside CNN-based approaches.

**Table 5:** Comparison of the present review with previous reviews on artificial intelligence and medical imaging.

Review	Scope of review	Main findings	Difference from the present review
Zhou et al. (2021)	Comprehensive review of deep learning in medical imaging across disease diagnosis, segmentation, image reconstruction, registration, and emerging AI technologies.	Reported substantial improvements in diagnostic performance of deep learning models while identifying challenges relating to interpretability, data quality, and clinical translation.	Focused broadly on all deep learning methods and medical imaging applications without specifically examining CNN deployment within resource-constrained healthcare settings.
Wang J. et al. (2021)	Review of transfer learning and deep learning approaches for medical image analysis and segmentation.	Highlighted transfer learning as an effective strategy for improving performance where labeled medical datasets are limited.	Primarily evaluated algorithmic optimization and transfer learning techniques, with limited consideration of implementation barriers, explainability, or deployment challenges in low-resource settings.
Egger et al. (2022)	Meta-review of deep learning applications in medicine.	Demonstrated high reported diagnostic accuracy across AI studies but identified methodological weaknesses including limited external validation, dataset bias, and poor reporting quality.	Examined deep learning generally across medicine and did not specifically synthesize CNN architectures, deployment feasibility, or implementation challenges relevant to LMICs.
Mienye et al. (2025)	Review of convolutional neural networks in medical image analysis.	Summarized CNN architectures, disease-specific applications, model performance, and emerging developments across multiple imaging modalities.	Focused primarily on technical advances in CNNs and diagnostic applications without specifically evaluating resource-constrained deployment, lightweight architectures, or healthcare system implementation.
Present scoping review	CNN applications for medical imaging in resource-constrained healthcare settings, synthesizing evidence on architectures, diagnostic performance, transfer learning, explainability, and deployment barriers.	Identified five major themes: diagnostic performance, lightweight CNN architectures, explainable AI, transfer learning for limited datasets, and implementation barriers affecting sustainable deployment in underserved healthcare environments.	Provides the first context-specific evidence map integrating CNN architecture, diagnostic performance, explainability, transfer learning, and real-world deployment challenges specifically within resource-constrained healthcare systems, thereby identifying implementation gaps and future research priorities for equitable AI adoption.

## Implications of the review

The findings of this scoping review have important implications for clinical practice, research, health systems strengthening, and future artificial intelligence implementation within resource-constrained healthcare settings. The evidence suggests that CNNs possess considerable potential to support diagnostic decision-making where shortages of radiologists, pathologists, and laboratory specialists limit access to timely healthcare services. The identification of lightweight architectures and transfer learning approaches indicates that AI-assisted medical imaging may become increasingly feasible in low-resource environments with limited computational capacity. Furthermore, the findings emphasize the importance of integrating explainable and transparent AI systems to improve clinician trust and support informed clinical decision-making. From a research perspective, the review highlights the need for locally representative datasets and external validation studies to improve generalizability across diverse populations. Healthcare policymakers and technology developers may also consider prioritizing context-specific AI design approaches capable of functioning under infrastructural limitations commonly observed in underserved healthcare systems.

## Limitations of the review

Although this review provides a comprehensive mapping of available evidence relating to CNN applications in medical imaging within resource-constrained settings, certain considerations should be acknowledged when interpreting the findings. First, the review included only studies published in the English language, which may have limited inclusion of relevant evidence reported in other languages and regional journals. Secondly, as a scoping review, the primary objective was to broadly map evidence rather than formally assess methodological quality or risk of bias across included studies. Additionally, variations in study design, imaging modalities, datasets, and performance metrics across the included studies may have introduced heterogeneity that limits direct comparison of findings across different clinical contexts.

## Conclusion

This scoping review demonstrated that CNNs are increasingly being applied across a wide range of medical imaging applications and possess considerable potential to improve diagnostic support within resource-constrained healthcare environments. Evidence across the included studies indicated that CNN systems consistently achieved strong diagnostic performance across multiple disease conditions including tuberculosis, pneumonia, malaria, skin cancer, and other imaging-based clinical applications. The review further identified lightweight architectures, transfer learning strategies, and explainability approaches as important developments supporting the feasibility of AI implementation within low-resource settings. However, despite encouraging advances in predictive performance, important challenges remain regarding infrastructure limitations, algorithmic bias, insufficient dataset diversity, limited external validation, and practical deployment readiness. The findings suggest that future research should move beyond performance optimization alone and increasingly prioritize contextual adaptation, locally representative datasets, explainable systems, and real-world implementation studies to ensure sustainable and equitable integration of AI-assisted medical imaging technologies into healthcare systems serving underserved populations.
