# Supplementary material for: Convolutional neural networks for medical imaging in resource-constrained settings: a scoping review of architectures, performance, and deployment challenges
Source: Front Artif Intell. 2026 Sep 9;9:1894126. doi: 10.3389/frai.2026.1894126 (PMC13597720; doi:10.3389/frai.2026.1894126)
Supplement: Supplementary file 1 [file Supplementary_File_1.pdf]

Appendix A1: Detailed Search Strategy for PubMed

Search Step	Search Query	Search Results (n)
#1	"Convolutional Neural Networks"[MeSH] OR "CNN" OR "Deep Convolutional Neural Network" OR "DCNN"	28,941
#2	"Medical Imaging"[MeSH] OR "Diagnostic Imaging" OR "Image Interpretation, Computer-Assisted" OR "Medical Image Analysis"	59,278
#3	"Resource-Limited Settings" OR "Low-resource settings" OR "Underserved healthcare" OR "Developing countries" OR "LMICs"	17,516
#4	"Tuberculosis" OR "Pneumonia" OR "Malaria" OR "COVID-19" OR "Skin cancer"	186,334
#5	#1 AND #2	2,284
#6	#5 AND #3	834
#7	#6 AND #4	486
#8	Filters: English language, Human studies, Peer-reviewed	**432**

Total records retrieved from PubMed = 432

Appendix A2: Detailed Search Strategy for Scopus

Search Step	Search Query	Search Results (n)
#1	TITLE-ABS-KEY ("Convolutional Neural Network" OR "CNN" OR "Deep Learning")	44,936
#2	TITLE-ABS-KEY ("Medical imaging" OR "Diagnostic imaging" OR "Image classification" OR "Computer-aided diagnosis")	63,547
#3	TITLE-ABS-KEY ("Low-resource settings" OR "Resource-constrained settings" OR "LMICs" OR "Developing countries")	14,839
#4	TITLE-ABS-KEY ("Tuberculosis" OR "Pneumonia" OR "Malaria" OR "COVID-19")	154,889
#5	#1 AND #2	2,779
#6	#5 AND #3	817
#7	#6 AND #4	639
#8	Filters: English, peer-reviewed articles	**582**

Total records retrieved from Scopus = 582

Appendix A3: Detailed Search Strategy for Web of Science

Search Step	Search Query	Search Results (n)
#1	TS=("Convolutional Neural Network" OR "CNN" OR "Deep Learning")	38,423
#2	TS=("Medical Imaging" OR "Diagnostic Imaging" OR "Image Analysis")	48,118

#3	TS=("Resource-constrained settings" OR "Low-resource settings" OR "LMICs")	10,277
#4	TS=("Tuberculosis" OR "Pneumonia" OR "Malaria" OR "Skin cancer")	119,447
#5	#1 AND #2	1,876
#6	#5 AND #3	659
#7	#6 AND #4	451
#8	Filters: Article, English	**414**

Total records retrieved from Web of Science = 414

Appendix A4: Detailed Search Strategy for IEEE Xplore

**Search Step**	**Search Query**	**Search Results (n)**
#1	("Convolutional Neural Networks" OR "CNN" OR "Deep Learning")	33,751
#2	("Medical Imaging" OR "Image Analysis" OR "Disease Classification")	29,635
#3	("Low-resource settings" OR "Resource-constrained settings" OR "LMICs")	7,964
#4	("Tuberculosis" OR "Malaria" OR "Pneumonia")	42,335
#5	#1 AND #2	1,923
#6	#5 AND #3	404
#7	#6 AND #4	319
#8	Filters: Journals, English language	**291**

Total records retrieved from IEEE Xplore = 291

Appendix A5: Detailed Search Strategy for ScienceDirect

**Search Step**	**Search Query**	**Search Results (n)**
#1	("Convolutional Neural Networks" OR "CNN" OR "Deep Learning")	52,681
#2	("Medical Imaging" OR "Diagnostic Imaging" OR "Computer-Aided Diagnosis")	67,114
#3	("Resource-constrained settings" OR "Low-resource settings" OR "LMICs" OR "Developing countries")	18,491
#4	("Tuberculosis" OR "Pneumonia" OR "Malaria" OR "COVID-19")	173,419
#5	#1 AND #2	2,478
#6	#5 AND #3	1,042
#7	#6 AND #4	833
#8	Filters: Research articles, English language	**814**

Total records retrieved from ScienceDirect = 814
